# Regional Brain Atrophy and Functional Connectivity Changes Related to Fatigue in Multiple Sclerosis

**DOI:** 10.1371/journal.pone.0077914

**Published:** 2013-10-22

**Authors:** Álvaro Javier Cruz Gómez, Noelia Ventura Campos, Antonio Belenguer, César Ávila, Cristina Forn

**Affiliations:** 1 Departament de Psicología Bàsica, Clínica i Psicobiología, Universitat Jaume I, Castelló de la Plana, Castellón, Spain; 2 Servicio de Neurología, Hospital General de Castellón, Castelló de la Plana, Castellón, Spain; Beijing Normal University, China

## Abstract

Fatigue is one of the most frequent symptoms in multiple sclerosis (MS), and recent studies have described a relationship between the sensorimotor cortex and its afferent and efferent pathways as a substrate of fatigue. The objectives of this study were to assess the neural correlates of fatigue in MS through gray matter (GM) and white matter (WM) atrophy, and resting state functional connectivity (rs-FC) of the sensorimotor network (SMN). Eighteen healthy controls (HCs) and 60 relapsing-remitting patients were assessed with the Fatigue Severity Scale (FSS). Patients were classified as fatigued (F) or nonfatigued (NF). We investigated GM and WM atrophy using voxel-based morphometry, and rs-FC changes with a seed-based method and independent component analysis (ICA). F patients showed extended GM and WM atrophy focused on areas related to the SMN. High FSS scores were associated with reductions of WM in the supplementary motor area. Seed analysis of GM atrophy in the SMN showed that HCs presented increased rs-FC between the primary motor and somatosensory cortices while patients with high FSS scores were associated with decreased rs-FC between the supplementary motor area and associative somatosensory cortex. ICA results showed that NF patients presented higher rs-FC in the primary motor cortex compared to HCs and in the premotor cortex compared to F patients. Atrophy reduced functional connectivity in SMN pathways and MS patients consequently experienced high levels of fatigue. On the contrary, NF patients experienced high synchronization in this network that could be interpreted as a compensatory mechanism to reduce fatigue sensation.

## Introduction

Fatigue is defined as an overwhelming sense of tiredness, lack of energy, or exhaustion [1]. It is one of the most disabling symptoms in patients with multiple sclerosis (MS), affecting between 50% and 80% of them [2]. Fatigue experienced by patients with MS seems to be distinct from fatigue in healthy individuals or those with other neurological diseases [3], and carries a major physical and psychological burden [4]. The pathogenesis of fatigue in MS is not fully understood, likely due to the multifactorial etiology of fatigue in these patients [4]. 

Magnetic resonance imaging (MRI) studies have contributed to describe possible factors related to this disabling symptom. Although initial studies yielded conflicting results [5,6], recent reports have described an association between fatigue and higher lesion load as well as gray matter (GM) atrophy [7–11]. Regarding whether or not lesion load or GM atrophy in specific brain areas could play a role in the occurrence and clinical characteristics of fatigue, volumetric studies have described alterations in frontal motor areas and certain subcortical areas, such as the thalamus and basal ganglia, that may be especially relevant [12–14]. Accordingly, it has been proposed that MS lesions at circuits relating to motor and premotor functions, and their afferent and efferent connections with several subcortical areas, could be the main substrate of fatigue in this clinical population [13]. Evidence for such dysfunction of the motor networks has been also provided by functional MRI (fMRI) studies in which fatigued patients showed increased activation in those circuits while performing a motor task [15,16]. More recently, these studies have been devoted to exploring patterns of spontaneous and synchronized activity in different brain regions during resting-state fMRI. Activity of these resting-state networks (RSNs) resembles that of neuroanatomical networks involved in specific sensory, motor, and cognitive functions, and it is thought that this activity does not only reflect intrinsic brain functional organization but also serves to stabilize brain ensembles [17]. One of the RSNs is the sensorimotor network (SMN), which is related to functional activity in the sensorimotor system and is therefore a network that may be relevant to the emergence of fatigue-related symptoms. Although activity of the SMN has been studied in MS patients in relation to their motor impairment [18–20], there are no studies addressing a possible relationship between SMN synchronization and fatigue in these patients. 

We hypothesized that variability in the organization and activity of motor networks could be related to the fatigue symptoms observed in MS patients. To test this hypothesis, we applied VBM and connectivity analyses on the RSNs trying to: 1) observe possible differences between fatigued (F) and nonfatigued (NF) patients compared to healthy controls (HCs) in GM and white matter (WM) volume, and their possible relationship with scores on an assessment of fatigue; 2) evaluate if the relationship between structural damage in motor areas and functional connectivity alterations within the SMN may account for fatigue; and 3) discern possible differences among the three groups of the study in intrinsic resting-state functional connectivity (rs-FC) of the SMN. 

## Materials and Methods

### Participants

We recruited 60 relapsing-remitting MS patients diagnosed according to the McDonald criteria [21] and 18 HCs with no previous history of neurological dysfunction. Recruited patients had no history of neurological or psychiatric disorders other than MS, and no receiving steroids-based treatment or experiencing a clinical relapse in the previous 2 months or other concomitant therapy as antidepressant or therapy for fatigue. MS disability was evaluated with the Expanded Disability Status Scale [22] and fatigue was assessed using the Fatigue Severity Scale (FSS) [23]. Depression symptomatology was assessed with the Chicago multiscale depression inventory (CMDI). According to previous studies [10],, patients who obtained an FSS score of greater than or equal to 4 were considered F (*N* = 32), whereas those with an FSS score of less than 4 were considered NF (*N* = 28). 

#### Standard protocol approvals and patient consents

Approval was received from the local ethical standards committee on human experimentation of Universitat Jaume I and Hospital General and written informed consent was obtained from all subjects. 

### MRI Acquisition

The fMRI session consisted of resting-state data acquired on a 1.5 T scanner (Siemens Avanto, Erlangen, Germany). A total of 270 volumes were recorded over 9 minutes using a gradient-echo T2*-weighted echo-planar imaging sequence (TR/TE = 2000/30 ms, matrix = 64 x 64 x 30, voxel size = 3.5 x 3.5 x 4.02 mm, flip angle = 90°). Participants were instructed to keep their eyes closed, stay motionless and relaxed without falling asleep, and think of nothing in particular. Prior to the functional sequences, a sagittal high-resolution three-dimensional (3D) T1-weighted sequence was acquired (TR = 11 ms, TE = 4.9 ms, FOV = 24 cm, matrix = 256 x 224 x 176, voxel size = 1 x 1 x 1 mm).

### Lesion Volume and Brain Volume Measurements

GM and WM volumes, and intracranial volume (ICV) for every participant were obtained from 3D T1 images using the unified segmentation approach of Statistical Parametric Mapping (SPM) 8 software (Wellcome Trust Centre for Neuroimaging, London, UK). 

In all patients, T1-hypointense lesions were manually identified and were semiautomated painted as regions of interest (ROIs) with the Jim software (Version 5.0, Xinapse Systems, Northants, UK; http://www.xinapse.com) using the T1 sagittal images converted to axial. We used the T1 acquired images as previously described by Ceccarelli et al., (2012) [24] to be more precise detecting the lesions because in this case, 176 images were acquired. Lesion masks for each patient were created (transforming the ROIs into independent images) using the same Jim software and then binarized using ImCalc module in SPM v.8. We also created lesion probability maps from all the 3D binary masks using a threshold of 0.1, showing voxels in which a lesion was presented in at least 10% of the patients [25]. 

After this, we used Lesion Filling tool [26] of the FMRIB Software Library (FSL, www.fmrib.ox.ac.uk/fsl) [27]. This tool takes the binary lesion masks together with the T1 sagittal images and creates a new structural image with lesion areas “filled” with intensities of neighborhood white matter voxels. These new images were used to improve the segmentation process of VBM analysis to obtain a more accurate GM and WM volumes.

Then images were reoriented along the anterior-posterior commissure. Optimized VBM was performed on the 3D lesion filled images using Diffeomorphic Anatomical Registration Through Exponential Lie Algebra (DARTEL) included in SPM v.8.The reoriented images were segmented into GM, WM, and cerebrospinal fluid images in native space, and then generated by a rigid transformation. The resolution of the aligned images was specified as 1.5 x 1.5 x 1.5 mm^3^. The study-specific GM and WM templates were then created by the aligned images from all patients and controls. The procedure began with the generation of an original template, computing the average of all aligned data, followed by the first iteration of the registration for each participant in turn. Thus, a new template was created and the second iteration began. After six iterations, the template was generated, which was the average of the DARTEL registered data. During iterations, all images were warped to the template, yielding a series of flow fields and parameterized deformations, which were employed in the modulation step. Since this was processed in native space, it was necessary to transform all the normalized, modulated data into Montreal Neurological Institute (MNI) space. After the space transformation, all these images were smoothed using an isotropic Gaussian kernel with 8-mm full width at half maximum.

The distribution of brain atrophy and differences among groups were assessed using an ANCOVA for GM and WM, including age, gender, and ICV as nuisance covariates. Finally, linear regression analyses were used to assess the relationship between WM and GM atrophy and FSS scores in all MS patients as a whole but also in F and NF patients separately.

 For all analyses, we used a family-wise error correction for multiple comparisons at the cluster level (*p* < .05) determined by Monte Carlo simulations conducted with the AlphaSim utility in REST software (http://www.restfmri.net/), implementing a voxel-wise threshold of *p* < .001 in combination with a cluster-size criterion of 132 voxels for GM and 146 voxels for WM [28].

### RSN Analysis

The rs-FC analysis was performed with two methods: a seed-based rs-FC method using the GM atrophy in sensorimotor areas as seeds to study whether the structural change causes a functional change in these areas, and independent component analysis (ICA) to show the differences in rs-FC of the network associated with the SMN. Both methods required specific preprocessing that is described in the supplementary material.

### Seed-Based Rs-FC Analysis

We tested the relationship between GM atrophy and rs-FC using regions of interest obtained in the VBM results (specifically in the contrast between F patients and HCs) in areas that we considered part of the sensorimotor cortex that includes the bilateral supplementary motor area (SMA), lateral primary motor cortex (PMC), and bilateral thalamus (see VBM results and seed-based Rs-FC results). After preprocessing, these regions of interest were resliced to the same normalization space of rs-fMRI data for subsequent rs-FC analysis. We computed voxel-wise rs-FC maps to disentangle the networks evoked by the seed regions. This method allowed us to study the rs-FC (Pearson’s correlation) of the seed region with all other voxels in the whole brain for each participant. Individual r-maps were normalized to z-maps using Fisher’s Z transformation. A one-sample t-test for each region was performed by entering the z-maps to detect brain areas showing significant rs-FC across participants and to obtain functional connectivity maps for each group (see seed-based Rs-FC results). To examine the changes in rs-FC between groups we performed a between-subjects ANOVA. Finally, we examined a possible relationship between rs-FC and FSS scores using a regression analysis. To avoid a possible confounding effect due to excessive head motion [29], we calculated the mean FD [30] using DPARSF (see Supplementary information), which was used as a covariate in each and all ANOVAs used to evaluate between-groups comparisons. We also performed an ANOVA with the mean FD to assess possible differences in head motion between the three groups. 

### Rs-FC Analysis of RSNs

 Intrinsic activity measured with rs-fMRI is organized in a limited number of RSNs and this finding has been replicable across studies [31,32]. To obtain these predefined RSNs, we performed an ICA for all participants, implemented in Group ICA of fMRI Toolbox (GIFT) software (http://icatb.sourceforge.net) [33]. A group-level spatial ICA using an infomax ICA algorithm [34] was utilized to extract 20 independent components (ICs). We identified one IC as the SMN (see Results and supplementary material) and used this RSN to show the differences in rs-FC of the network associated with motor areas among groups with a between-subjects ANOVA.

 All rs-FC results were presented using family-wise error corrected for multiple comparisons at the cluster level (*p* < .05) determined by whole-brain Monte Carlo simulations conducted with AlphaSim implemented in REST (voxel-wise threshold of *p* < .005; cluster-size criterion of 12 voxels).

## Results

Table 1 summarizes demographic, clinical, and MRI characteristics of each group of participants. ICV was significantly larger in HCs than in both subgroups of MS patients. On the other hand, F patients had higher scores in the EDSS scale as well as in all CDMI subscales. F patients exhibited higher scores than HC in all CDMI scales except in that measuring vegetative symptoms. In addition, no significant differences head motion values between groups (p > 0.10) were observed. FSS and EDSS scores were not significantly correlated (F patients: rho=0.062, p=0.73; NF patients: rho= 0.16, p=0.39). 

**Table 1:** Main demographic, clinical and MRI characteristics from all participants.

	**HC n=18**	**NF n= 28**	**F n=32**	**HC vs. NF**	**HC vs. F**	**NF vs. F**
Gender (male / female)	10 / 8	10 / 18	11 / 21	n.s.	n.s.	n.s.
Age mean (SD) [range]	31.06 (5.67) [22-44]	34.96 (5.87) [20-44]	37.72 (5.90) [22-47]	n.s.	.001	n.s.
Years of evolution disease mean (SD) [range]		5.14 (3.69) [1-14]	7.44 (5.15) [1-14]			n.s.
FSS mean (SD)		2.21 (0.96)	5.6 (0.85)			.000
EDSS mean (SD) [range]		1.96 (1.20) [0-5]	3.20 (1.68) [1-6]			.002
CMDI Mood Scale mean (SD)	22 (7.44)	21.21 (7.04)	32.14 (12.11)	n.s.	.003	.000
CMDI Evaluative Scale mean (SD)	17 (3.76)	17.14 (4.2)	25.41 (10.1)	n.s.	.001	.000
CMDI Vegetative Scale mean (SD)	32.11 (8.67)	25.75 (7.21)	38.59 (10.52)	n.s.	n.s.	.000
CDMI Total Score mean (SD)	71.11(16.02)	64.11(16.23)	96.14 (29.90)	n.s.	.002	.000
T1 LV (ml) mean (SD)		3.16 (3.97)	6.03 (14.02)		n.s.	n.s.
ICV ml mean.(SD)	1261.24 (102.63)	1141.34 (121.98)	1101.16 (144.74)	.011	.000	n.s.

Abbreviations: HC = healthy controls; NF = non fatigued; F = fatigued; CMDI = Chicago multiscale depression inventory; LV= lesion volume; ml = milliliters: intracranial volume=ICV; n.s= non significant

### VBM Results

**Figure 1:**
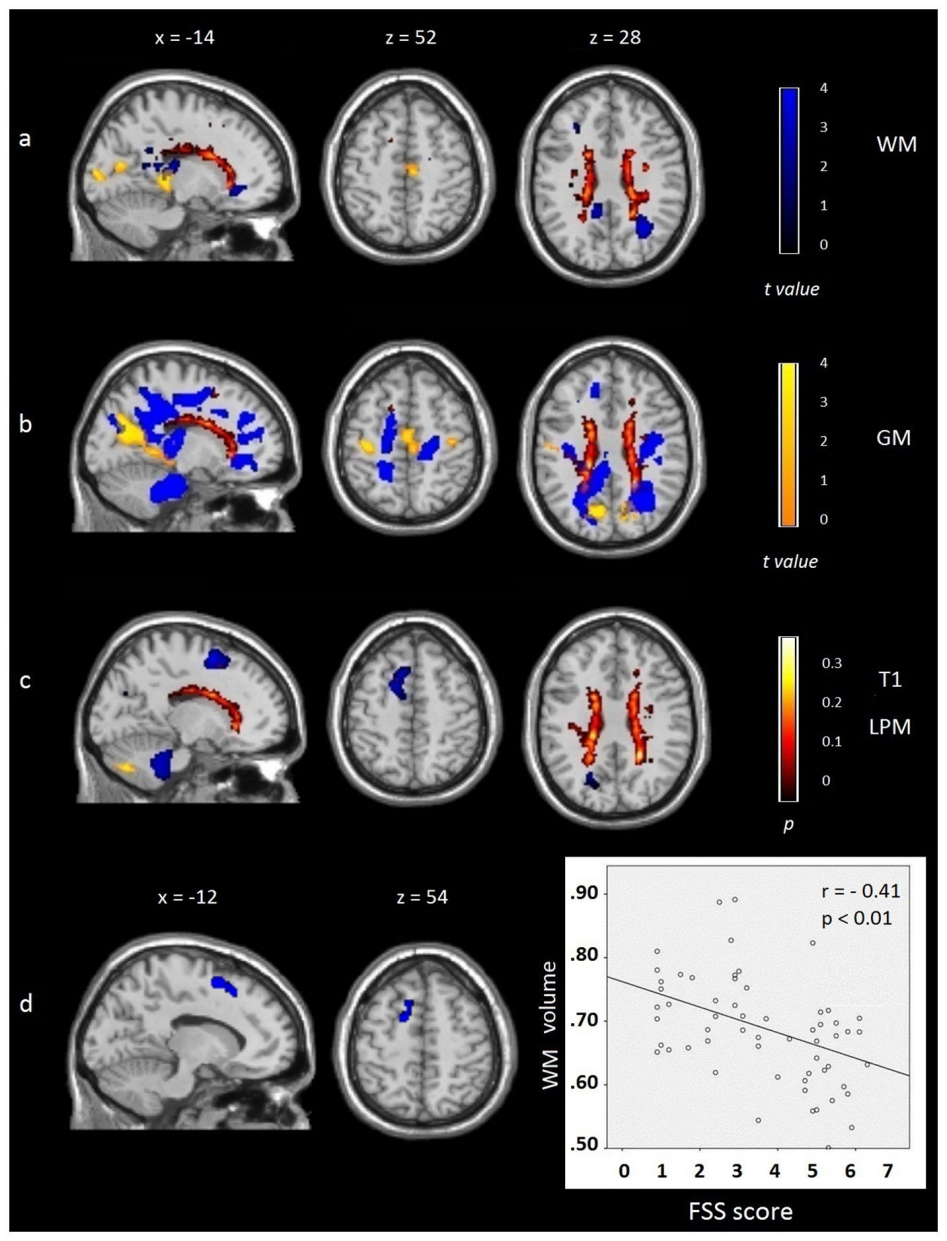
Overlay map of gray matter (GM; t values in yellow) and white matter (WM; t values in blue) atrophy distribution and lesion probability maps (t values in red) presented using family-wise error corrected for multiple comparisons (**Monte Carlo *p* < .001)**. A) Nonfatigued patients compared to healthy controls. B) Fatigued patients compared to healthy controls. C) Fatigued patients compared to nonfatigued patients. D) Correlation between high Fatigue Severity Scale (FSS) scores and reduction of WM volume in the left supplementary motor area in all MS patients. Images are presented in neurological convention. See text for further details.

Compared to HCs, NF patients exhibited a higher degree of GM atrophy in the right paracentral gyrus (SMA), different areas of the bilateral temporal and occipital lobes, the right precuneus, and bilateral thalamus (see Figure 1A). Compared to the HC group, F patients exhibited GM atrophy in the paracentral gyrus (SMA), bilateral precentral gyrus (PMC), bilateral occipital lobe, precuneus, and posterior cingulate gyrus (see Figure 1B). Differences between both patient subgroups were observed in the left cerebellum (MNI -11 -74 -39, k = 379, t = 4.02) where F patients showed a reduction of GM volume compared to NF patients (see Figure 1C). No differences were observed in the reverse contrasts.

In comparison to the HC group, WM structural changes in NF patients achieved statistical significance in several WM areas of the bilateral frontal lobe, right middle cingulate gyrus, bilateral posterior cingulate gyrus, bilateral temporal and occipital lobes, around the left thalamus, and bilateral corpus callosum (see Figure 1A). Compared to HCs, F patients showed WM alterations that extended into a larger number of brain regions in the frontal (including the motor areas and insula), temporal, occipital, and parietal lobes. F patients also showed WM atrophy around the bilateral thalamus, bilateral corpus callosum, and WM of cingulate gyrus (anterior, middle, and posterior parts), and WM of the bilateral brainstem, and cerebellum (see Figure 1B). On the other hand, compared to the NF group, F patients showed WM atrophy in left frontal areas (k = 1381) that included the left medial frontal gyrus of the SMA (MNI -9 5 55, t = 4.55), left superior frontal gyrus (MNI -14 14 55, t = 3.99), left precuneus (MNI -18 -71 24, t = 3.6, k = 273), bilateral brainstem (right, MNI 3 -24 -40; left, MNI -2 -24 -40), and WM of the left cerebellum (MNI -12 -44 -44; k = 2976; see Figure 1C). No differences were observed in the reverse contrasts. For further details see Table S1 and Table S2. 

The regression analysis showed that high FSS scores were associated to reduced WM volumes (that is, with a significant degree of atrophy) in the left SMA (MNI -11 -20 50 r=-.41) see Figure 1D. 

### Seed-Based Rs-FC Results

**Figure 2:**
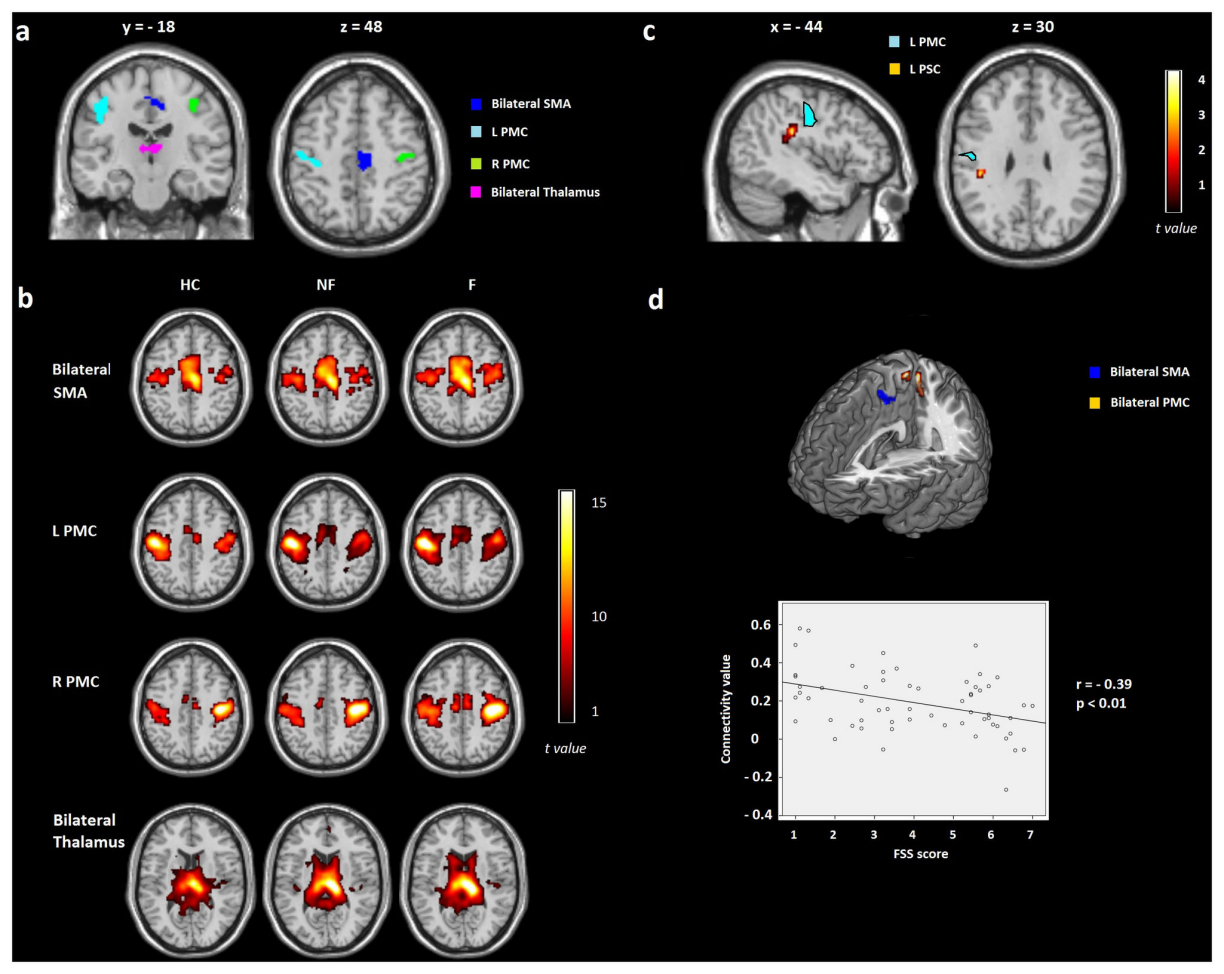
Results of the seed analysis presented using family-wise error corrected for multiple comparisons (Monte Carlo *p* < .005, k = 12). A) Regions of interest obtained from the volumetric results that were part of the sensorimotor network: bilateral supplementary motor area (SMA), left primary motor cortex (LPMC), right primary motor cortex (RPMC), and bilateral thalamus. B) Maps evoked with a seed region analysis for each group of the study: healthy controls (HC), nonfatigued patients (NF), and fatigued patients (F). C) ANOVA results of the seed analysis showing decreased of connectivity between the LPMC and left primary somatosensory cortex (LPSC) in MS patients compared to HC; D) High Fatigue Severity Scale (FSS) scores associated with decreased connectivity between the bilateral SMA and bilateral PMC in all MS patients. See text for further details.

We selected four regions of interest within sensorimotor brain areas that differed between the F group and controls in the VBM analyses (see Figure 2A) and we used them as seeds to perform rs-FC analyses. The functional connectivity maps obtained for each group in each seed appear in Figure 2B. Results of comparisons among groups only showed that MS patients presented decreased of rs-FC between the left PMC (MNI -47 -15 41; t= 4.15, k= 961) and left primary somatosensory cortex (MNI -42 30 27; t= 4.22, k= 41) compared to controls (see Figure 2C). No other differences among groups were observed. 

Seed regression analysis within MS patients using FSS scores as a covariate of interest showed that higher FSS scores were associated with lower rs-FC between the bilateral SMA (MNI 8 -21 48) and bilateral PMC (MNI 3 -39 66 and -6 -39 66; *r* = -.39; see Figure 2D). 

### Results of Rs-FC Analysis of RSNs

**Figure 3:**
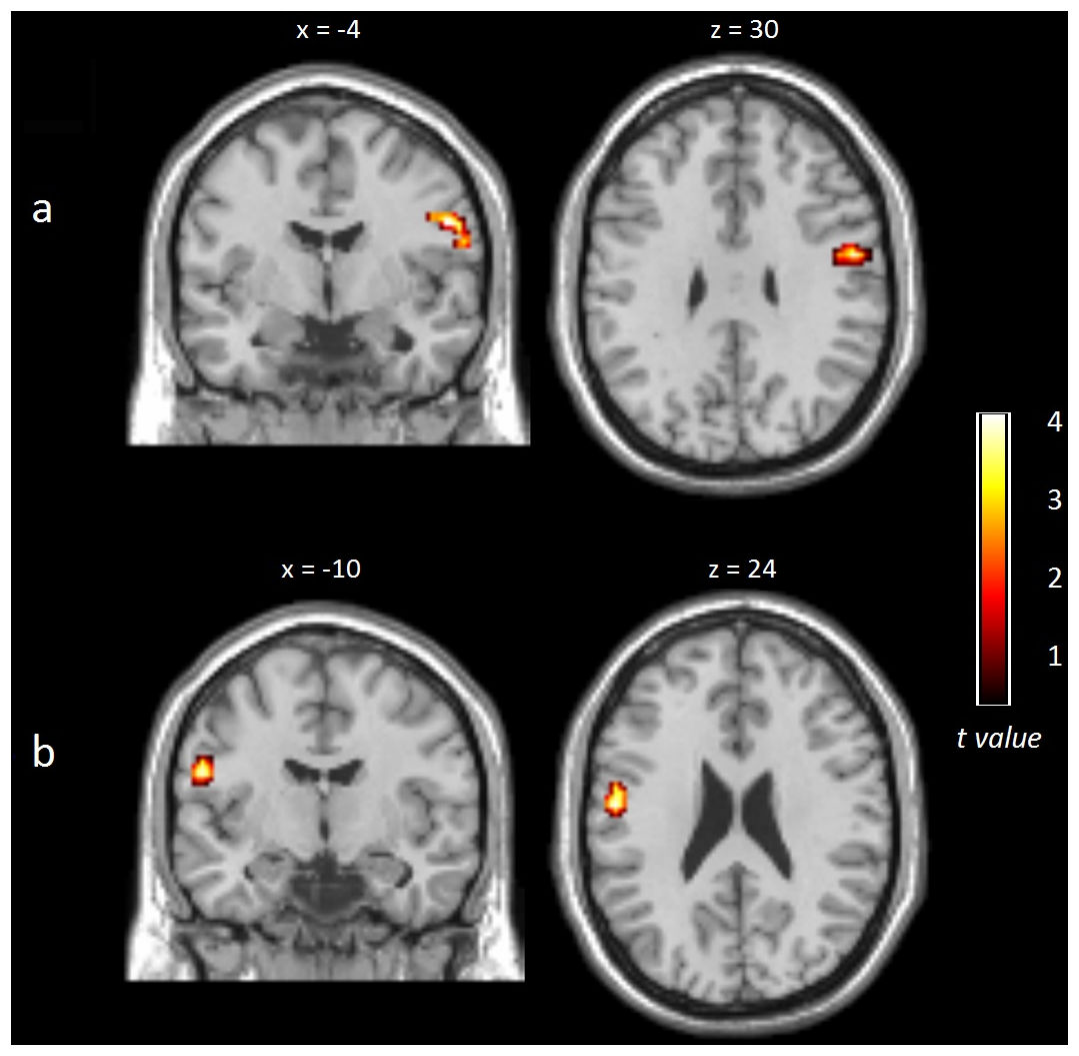
Results on the sensorimotor resting-state network presented using family-wise error corrected for multiple comparisons (Monte Carlo *p* < .005, k = 12). A) Increased synchronization in nonfatigued patients (NF) compared to healthy controls (HC) observed in the right precentral gyrus. B) Increased synchronization in NF patients compared to fatigued (F) patients observed in the left postcentral gyrus. See text for further details.

The RSN of intrinsic connectivity was constructed using ICA, identifying 8 RSNs (illustrated Figure S1 and Table S3). Based on networks reported in previous studies [31,32,35–37], we classified 8 RSNs that are reported in the Supplementary Material. The SMN was selected as the network of interest and comprised the precentral and postcentral gyri, medial frontal gyri, SMA, PMC, thalamus, and caudate of the basal ganglia and cerebellum. Comparison of groups using ANOVA demonstrated that NF patients showed significantly greater rs-FC of the right precentral gyrus and PMC (MNI 63 -4 19 t= 2.87, k= 19) than controls. Compared to F patients, NF also showed increased rs-FC in the left precentral gyrus, in this case associated with the premotor cortex (MNI -54 -10 22 t= 3.77, k=24); see also Figure 3). No other differences among groups were observed.

## Discussion

The purpose of the present study was to investigate the neural correlates of fatigue in people with MS. Our results indicate that F patients presented more extensive GM and WM atrophy in areas related to motor functions such as the SMA, PMC, cerebellum, and brainstem. Another important finding was that fatigue scores were also associated with rs-FC levels in the pathways connecting these brain areas involved in processing sensory and motor information Thus, F patients displayed decreased levels of rs-FC in these pathways while NF patients displayed increased levels.

The results provided by the VBM analysis showed that F and NF patients presented GM atrophy in the SMA, but GM volume reduction also extended to the PMC as well as to the posterior part of the cingulate gyrus and cerebellum in F patients. WM atrophy was more widespread than GM atrophy, but differences between both patient subgroups were also found in the SMA and other areas of the SMN. Thus, our findings confirm the important role of atrophy at the frontoparietal SMN in the perception of fatigue. This putative relationship had already been described in previous studies [8,10,38,39] and now finds further support from the significant correlation between FSS scores and WM volume reduction in the left SMA, observed in our study. We additionally found that F patients presented a reduction of GM and WM volume in other areas involved in sensory and motor functions such as the cerebellum and brainstem. 

The pathogenesis of fatigue in MS is not well understood, probably because different factors may influence this symptom [11]. Nevertheless, it seems reasonable to suggest that atrophy localized in frontoparietal sensory and frontoparietal motor networks could produce retrograde degeneration of axons that results in dysfunctional connections within the SMN. We tested this hypothesis by analyzing rs-FC within four brain areas belonging to the SMN at which F patients exhibited a significant degree of atrophy. Rs-FC between the left PMC and left primary somatosensory cortex was reduced in F and NF patients compared to HCs. Further, when using FSS scores as a covariate of interest, we identified an inverse correlation between fatigue levels and rs-FC between the bilateral SMA and bilateral PMC. The SMA and PMC are involved in processes related to the control of movement and previous studies have described in the SMA higher activity in F than in NF patients during the execution of motor tasks [40].

We extended our analysis of rs-FC to all pathways related to the SMN and not only to areas where GM atrophy was observed in F patients. Interestingly, we found that compared not only to F patients but also to HCs, NF patients showed significant increases of rs-FC between the right precentral gyrus and PMC as well as between the left precentral gyrus and premotor cortex. These data converge again with fMRI studies suggesting a relationship between MS fatigue and brain activity disturbances in different areas involved with sensorimotor functions. More specifically, Rocca et al., (2009) [16] showed that, while performing a complex motor task, MS patients with fatigue displayed decreased activation of similar areas where we observed decreased rs-FC, namely the precentral and postcentral gyri. In this way, and although we must interpret these results with caution, the increased rs-FC observed in NF patients with respect to the other groups (particularly the HC group) may reflect a compensatory mechanism associated with subclinical fatigue.

It is important to note that previous studies have suggested that fatigue in MS patients is also related to structural abnormalities of the basal ganglia and thalamus as well as their extensive interconnections with other brain areas [12,39]. Similarly, another study [16] observed increased activation of the basal ganglia in fatigued MS patients performing a complex task. According to these precedents, we expected to find differences between F and NF patients in atrophy, and also rs-FC in the basal ganglia or thalamus as well as in the cortico-basal ganglia-thalamocortical loops. Although we observed more atrophy in the thalamus of F than NF patients, these differences did not reach statistical significance. Moreover, we did not observe volumetric or rs-FC differences in the basal ganglia. 

There is a possible limitation in our study that might be worth considering here, which is the fact that F patients displayed higher EDSS scores than NF patients. However, we do not think that physical disability might account for the fatigue differences found between both MS patients subgroups. This conclusion is based in the fact that we did not find any correlation between FSS and EDSS scores in any of these subroups of MS patients. Future studies should be addressed to observe a possible association between sensoriomotor alterations and fatigue in a wider sample of MS with different EDSS scores. 

In summary, the present results are unprecedented in showing a relationship between fatigue and rs-FC changes related to atrophy. We observed that reduced rs-FC also extended to other areas of the SMN where no differences in atrophy were observed between F and NF patients but that might be responsible for poor integration of the sensory and motor pathways. Fatigue sensation seems to be related to decreased synchronization between the right precentral gyrus and PMC as well as between the left precentral gyrus and premotor cortex. Interestingly, enhanced rs-FC in this network was observed in MS patients reporting low levels of fatigue. This enhanced connectivity may act as a compensatory and adaptive functional change necessary to maintain “normal” vigor sensation in some MS patients. 

## Supporting Information

Material S1 **Resting state networks (RSN) analysis.**
(DOC)

Figure S1 **Spatial maps of eight resting state networks (RSNs) construct using independent component analysis (ICA).**
(TIFF)

Table S1 **Areas showing differences in gray matter volume (GM) between groups according to fatigue.** Results are presented at corrected multiple comparisons (Monte-Carlo, p<0.001), k=132. Abbreviations: NF non fatigued patients, F fatigued patients, HC healthy controls, R right, L left, Supplementary motor area (SMA); Primary motor cortex (PMC).
(PDF)

Table S2 **Areas showing differences in white matter volume (WM) in between groups according to fatigue.** Results are presented at corrected multiple comparisons (Monte-Carlo, p<0.001), k=146. Abbreviations: NF non fatigued patients, F fatigued patients, HC healthy controls, R right, L left; Supplementary motor area (SMA).
(PDF)

Table S3 **Anatomical regions of Sensorimotor Network (SMN) identified on three groups using Independent Component Analysis (ICA).** Corrected at FWE p < 0.05. Abbreviations: HC = healthy controls; NF = non fatigued; F = fatigued; R = right; L = left; BA = Brodmann Area.
(PDF)
