# Supplementary material for: Regional Brain Atrophy and Functional Connectivity Changes Related to Fatigue in Multiple Sclerosis
Source: PLoS One. 2013 Oct 22;8(10):e77914. doi: 10.1371/journal.pone.0077914 (PMC3805520; doi:10.1371/journal.pone.0077914)
Supplement: Material S1 — Resting state networks (RSN) analysis. (DOC) [file pone.0077914.s001.doc]

**Resting state networks (RSN) analysis**

**Preprocessing for resting state functional connectivity (rs-FC)**

Rs-FC analysis was preprocessed using SPM v.8 software. Preprocessing included slice-timing correction for interleaved acquisitions using sinc interpolation and resampling with the middle (29th) slice in time as a reference point. Head motion correction, spatial normalization with resampling to 3 mm^3^ voxels, and spatial smoothing were performed with an isotropic Gaussian kernel of 4-mm full width at half maximum.

**Additional Preprocessing for seed-based Rs-FC**

We conducted additional preprocessing and rs-FC analysis using a toolkit of the Data Processing Assistant for Resting-State fMRI (DPARSF; http://www.restfmri.net; [1] through the following steps: 1) removing the linear trend in the time series; 2) temporally band-pass filtering (0.01–0.08 Hz) to reduce the effect of low-frequency drift and high-frequency noise [2,3]; and 3) controlling non-neural noise in the seed region time series [4]. Several sources of spurious variance were removed from the data through linear regression: six parameters obtained by rigid body correction of head motion, the global mean signal, WM signal, and cerebrospinal fluid signal.

**Rs-FC Analysis of RSNs using the ICA**

The minimum description length criterion was used to determine the number of ICs. Twenty iterations of ICA were performed using ICASSO to determine the reliability or stability of the ICA algorithm [5], and the best estimate (centrotype of the cluster) for each IC was used. The individual IC maps and time courses were computed using back-reconstruction based on aggregate ICs and the results from the data reduction step [6]. RSNs were classified by visually inspecting the aggregate spatial maps (discarding ICs associated with physiological artifacts) and average power spectra < 0.10 Hz; [3].

We identified 8 RSNs based on networks reported in previous studies [7–11]. We classified our 8 RSNs as follows: two networks corresponding to the visual system [3,12] represented by the (1) primary visual network, (2) lateral visual network (they include the inferior and superior occipital gyri, respectively); (3) the auditory network (AN), which includes the bilateral middle and superior temporal gyri, posterior insular cortex, superior temporal sulcus, and Heschl gyrus [13,14]; (4) default mode network (DMN) initially reported by Raichle *et al.* [15] and involving the posterior cingulated cortex/precuneus region, bilateral inferior parietal gyrus, middle temporal gyrus, and anterior cingulate gyrus; one network corresponding to motor and somatosensory functions (5) sensory-motor network (SMN) [2] including the precentral and postcentral gyri, the medial frontal gyri, SMA, primary motor cortex, thalamus and caudate of the basal ganglia and cerebellum; the (6) task positive network reminiscent of the dorsal attention network [16], and dorsolateral prefrontal cortices: the (7) left fronto-parietal network (LFPN), a left-lateralized network including the dorsolateral prefrontal cortex, ventrolateral prefrontal cortex, dorsomedial prefrontal cortex, parietal cortices, [16,17] and the (8) right fronto-parietal network (RFPN) associated with the central-executive network [18,19].

**References**

1 Chao-Gan Y, Yu-Feng Z. DPARSF: A MATLAB Toolbox for “Pipeline” Data Analysis of Resting-State fMRI. FNSYS 2010;**4**:13.

2 Biswal B, Yetkin FZ, Haughton VM, et al. Functional connectivity in the motor cortex of resting human brain using echo-planar MRI. Magn Reson Med 1995;**34**:537–41.

3 Lowe MJ, Mock BJ, Sorenson JA. Functional connectivity in single and multislice echoplanar imaging using resting-state fluctuations. NeuroImage 1998;**7**:119–32.

4 Fox MD, Snyder AZ, Vincent JL, et al. The human brain is intrinsically organized into dynamic, anticorrelated functional networks. P Natl Acad Sci USA 2005;**102**:9673–8.

5 Himberg J, Hyvärinen A, Esposito F. Validating the independent components of neuroimaging time series via clustering and visualization. NeuroImage 2004;**22**:1214–22.

6 Erhardt EB, Rachakonda S, Bedrick EJ, et al. Comparison of multi-subject ICA methods for analysis of fMRI data. Hum Brain Mapp 2011;**32**:2075–95.

7 Beckmann CF, DeLuca M, Devlin JT, et al. Investigations into resting-state connectivity using independent component analysis. Philos T Roy Soc B 2005;**360**:1001–13.

8 Damoiseaux JS, Rombouts SARB, Barkhof F, et al. Consistent resting-state networks across healthy subjects. P Natl Acad Sci USA 2006;**103**:13848–53.

9 Calhoun VD. Does the Brain Rest?: An Independent Component Analysis of Temporally Coherent Brain Networks at Rest and During a Cognitive Task. IEEE SSIAI 2008. 201–4.

10 Shehzad Z, Kelly AMC, Reiss PT, et al. The resting brain: unconstrained yet reliable. Cereb Cortex 2009;**19**:2209–29.

11 Smith SM, Fox PT, Miller KL, et al. Correspondence of the brain’s functional architecture during activation and rest. P Natl Acad Sci USA 2009;**106**:13040–5.

12 Grill-Spector K, Malach R. The human visual cortex. Annu Rev Neurosci 2004;**27**:649–77.

13 Seifritz E, Neuhoff JG, Bilecen D, et al. Neural processing of auditory looming in the human brain. Curr Biol 2002;**12**:2147–51.

14 Specht K, Reul J. Functional segregation of the temporal lobes into highly differentiated subsystems for auditory perception: an auditory rapid event-related fMRI-task. NeuroImage 2003;**20**:1944–54.

15 Raichle ME, MacLeod AM, Snyder AZ, et al. A default mode of brain function. P Natl Acad Sci USA 2001;**98**:676–82.

16 Corbetta M, Shulman GL. Control of goal-directed and stimulus-driven attention in the brain. Nat Rev Nerurosci 2002;**3**:201–15.

17 Dosenbach NUF, Fair DA, Miezin FM, et al. Distinct brain networks for adaptive and stable task control in humans. P Natl Acad Sci USA 2007;**104**:11073–8.

18 Koechlin E, Summerfield C. An information theoretical approach to prefrontal executive function. Trends Cogn Sci 2007;**11**:229–35.

19 Sridharan D, Levitin DJ, Menon V. A critical role for the right fronto-insular cortex in switching between central-executive and default-mode networks. P Natl Acad Sci USA 2008;**105**:12569–74.
