# Supplementary figures and images for: Regional Brain Atrophy and Functional Connectivity Changes Related to Fatigue in Multiple Sclerosis
Source: PLoS One. 2013 Oct 22;8(10):e77914. doi: 10.1371/journal.pone.0077914 (PMC3805520; doi:10.1371/journal.pone.0077914)

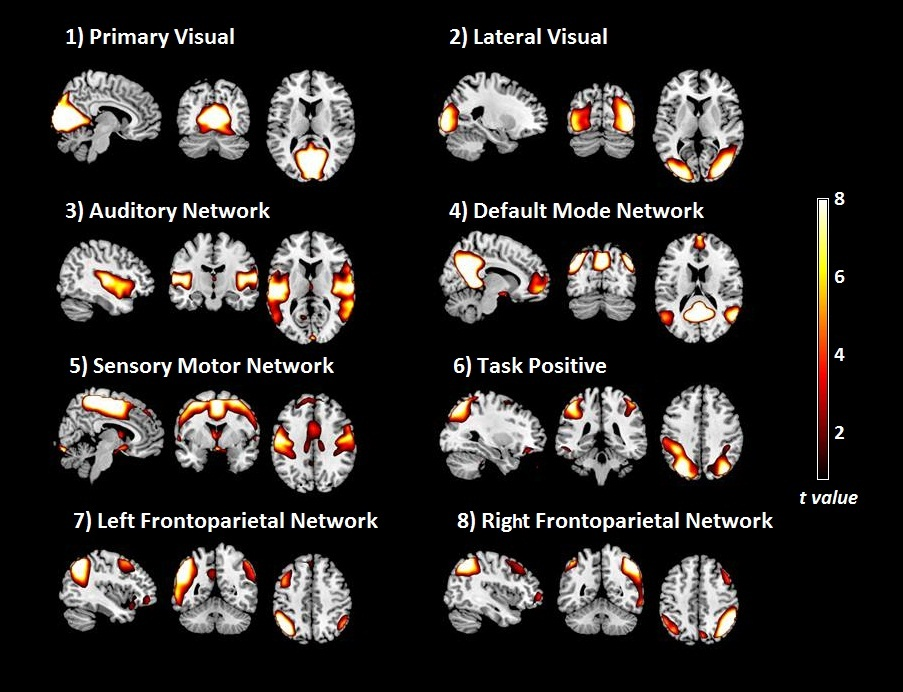

Supplement: Figure S1 — Spatial maps of eight resting state networks (RSNs) construct using independent component analysis (ICA). (TIFF) [file pone.0077914.s002.tiff]
