# Supplementary material for: Regional Brain Atrophy and Functional Connectivity Changes Related to Fatigue in Multiple Sclerosis
Source: PLoS One. 2013 Oct 22;8(10):e77914. doi: 10.1371/journal.pone.0077914 (PMC3805520; doi:10.1371/journal.pone.0077914)
Supplement: Table S1 — Areas showing differences in gray matter volume (GM) between groups according to fatigue. Results are presented at corrected multiple comparisons (Monte-Carlo, p<0.001), k=132. Abbreviations: NF non fatigued patients, F fatigued patients, HC healthy controls, R right, L left, Supplementary motor area (SMA); Primary motor cortex (PMC). (PDF) [file pone.0077914.s003.pdf]

Table S1

	NF vs. HC					F vs. HC				
**Regions of interest**	**Cluster size**	**t value**	**MNI coordinates**			**Cluste size**	**t value**	**MNI coordinates**		
			**x**	**y**	**z**			**x**	**y**	**z**
R Paracentral Lobe / SMA	135	3.88	9	-20	50	475	3.66	8	-21	48
L Paracentral Lobe / SMA	-	-	-	-	-		3.57	0	-11	56
R Medial Frontal Gyrus	-	-	-	-	-	209	3.84	5	-26	63
	-	-	-	-	-	181	3.55	6	33	-20
L Medial Frontal Gyrus	-	-	-	-	-	158	3.68	-2	57	-9
R Middle Cingulate	-	-	-	-	-	229	3.57	9	9	38
L Precentral Gyrus/PMC	-	-	-	-	-	961	4.15	-47	-15	41
R Precentral Gyrus /PMC	-	-	-	-	-	165	3.79	45	-17	47
L Superior Temporal Gyrus	-	-	-	-	-	148	3.81	-50	-21	1
R Temporal Lobe /Fusiform Gyrus	249	4.38	45	-56	-17	156	3.83	45	-54	-17
R Middle Temporal Gyrus	138	3.58	68	-30	-11	199	3.86	62	-35	-5
L Middle Temporal Gyrus	279	4.25	-57	41	0	-	-	-	-	-
R Inferior Temporal Gyrus	332	4.07	47	-66	-5	-	-	-	-	-
L Occipital Lobe / Lingual Gyrus	135	3.40	-23	-72	-9	398	4.71	-20	-71	9
R Occipital Lobe / Lingual Gyrus	-	-	-	-	-	174	4.24	11	-77	-3
L Superior Occipital Gyrus	-	-	-	-	-	2698	4.17	-17	-71	26
L Cuneus	316	3.70	-12	-73	14		4.51	-14	-74	18
	171	4.01	-17	-93	8		-	-	-	-
L Precuneus	-	-	-	-	-		3.96	-9	-60	14

L Hippocampus	-	-	-	-	-		3.54	-18	-35	-5
L Posterior Cingulate	-	-	-	-	-		3.70	-11	-57	10
R Posterior Cingulate	-	-	-	-	-	678	4.67	15	-57	11
R Thalamus	1496	4.13	17	-32	-2	543	3.99	8	-14	9
L Thalamus		4.15	-20	-30	6		3.75	-8	-17	9
R Cuneus	143	3.77	20	-75	32	539	4.81	20	-75	32
R Precuneus		3.67	18	-69	33		4.18	23	-74	24
