# Supplementary material for: Regional Brain Atrophy and Functional Connectivity Changes Related to Fatigue in Multiple Sclerosis
Source: PLoS One. 2013 Oct 22;8(10):e77914. doi: 10.1371/journal.pone.0077914 (PMC3805520; doi:10.1371/journal.pone.0077914)
Supplement: Table S2 — Areas showing differences in white matter volume (WM) in between groups according to fatigue. Results are presented at corrected multiple comparisons (Monte-Carlo, p<0.001), k=146. Abbreviations: NF non fatigued patients, F fatigued patients, HC healthy controls, R right, L left; Supplementary motor area (SMA). (PDF) [file pone.0077914.s004.pdf]

Table S2

	NF vs. HC					F vs. HC				
**Regions of interest**	**Cluster size**	**t value**	**x**	**y**	**z**	**Cluster size**	**t value**	**x**	**y**	**z**
L Temporal Lobe WM	433	4.01	-35	-66	11	229	3.94	-44	-8	-17
L Middle Occipital Gyrus WM		3.47	-34	-73	12	23124	3.53	-33	-72	12
R Middle Temporal Gyrus WM	-	-	-	-	-		4.42	40	-59	5
R Temporal Lobe WM	-	-	-	-	-		4.74	35	-65	3
R Thalamus WM	2558	3.41	11	-27	5		4.80	9	-24	3
R Parietal Lobe / Angular Gyrus WM		3.62	35	-59	35		-	-	-	-
R Inferior Parietal Lobule WM		-	-	-	-		4.64	35	-59	36
L Precuneus WM		-	-	-	-		5.05	-23	-71	17
R Middle Temporal Gyrus WM		3.33	40	-59	6		4.17	40	-59	6
R Posterior Cingulate WM		4.30	16	-54	15		4.29	16	-54	15
R Corpus Callosum WM		3.43	10	-46	12		3.71	10	-46	12
L Posterior Cingulate WM	644	3.35	-10	-48	15		3.75	-10	-48	15
L Corpus Callosum WM		3.33	-9	-45	17		3.67	-10	-46	12
L Thalamus WM	997	4.56	-8	-12	17		5.72	-8	-12	17
L Parietal Lobe WM	-	-	-	-	-		3.67	-28	-57	29
L Brainstem / Pons WM	-	-	-	-	-	5622	3.49	-2	-22	-25
R Brainstem / Pons WM	-	-	-	-	-		3.48	2	-22	-25
L Cerebellum WM	-	-	-	-	-		4.06	-21	-42	-36
R Cerebellum WM	-	-	-	-	-		3.26	14	-43	-43
R Postcentral Gyrus WM	-	-	-	-	-	4130	3.79	48	-26	35
R Precentral Gyrus WM	-	-	-	-	-		3.79	42	-8	29

R Paracentral Lobule / SMA	-	-	-	-	-		3.56	13	-28	52
R Cingulate Gyrus WM	154	3.59	14	-9	45	-	-	-	-	-
R Anterior Cingulate WM	376	3.59	16	38	-5	1477	3.29	16	41	10
R Middle Frontal Gyrus WM		3.49	24	39	-7		3.55	24	39	-7
R Medial Frontal Gyrus WM		3.29	16	48	3		3.69	16	48	3
R Insula WM	-	-	-	-	-		3.36	32	23	12
R Inferior Frontal Gyrus WM	-	-	-	-	-		3.72	33	25	7
L Inferior Frontal Gyrus WM	-	-	-	-	-	3116	3.95	-33	25	7
L Middle Frontal Gyrus WM	-	-	-	-	-		4.39	-26	45	-8
L Insula WM	-	-	-	-	-		4.44	-32	23	13
L Anterior Cingulate WM	210	3.69	-12	30	-9		4.02	-11	41	14
L Frontal Lobe WM	430	3.80	-29	32	27	-	-	-	-	-
L Superior Frontal Gyrus WM	-	-	-	-	-	239	4.02	-14	41	32
