# Supplementary material for: Regional Brain Atrophy and Functional Connectivity Changes Related to Fatigue in Multiple Sclerosis
Source: PLoS One. 2013 Oct 22;8(10):e77914. doi: 10.1371/journal.pone.0077914 (PMC3805520; doi:10.1371/journal.pone.0077914)
Supplement: Table S3 — Anatomical regions of Sensorimotor Network (SMN) identified on three groups using Independent Component Analysis (ICA). Corrected at FWE p < 0.05. Abbreviations: HC = healthy controls; NF = non fatigued; F = fatigued; R = right; L = left; BA = Brodmann Area. (PDF) [file pone.0077914.s005.pdf]

Table S3

	HC					NF					F				
**Anatomical Localisation**	**Cluster size**	**t value**	**x**	**y**	**z**	**Cluster size**	**t value**	**x**	**y**	**z**	**Cluster size**	**t value**	**x**	**y**	**z**
R Primary Motor Cortex (BA 4)	2408	14.95	15	-37	67	5458	15.95	51	-13	49	5454	14.65	36	-31	61
L Primary Motor Cortex (BA 4)		12.07	-27	-28	64		10.96	-33	-28	55		13.21	-30	-28	58
R Supplementary Motor Area (BA 6)		9.09	6	8	58		10.56	3	11	49		6.22	6	8	58
L Supplementary Motor Area (BA 6)		9.60	-3	8	49		7.86	-3	11	49		7.63	-3	11	49
R Premotor Cortex (BA 6)		16.86	18	-10	64		9.66	21	-10	64		6.16	21	-10	64
L Premotor Cortex (BA 6)		14.37	-36	-10	49		10.85	-36	-13	43		9.99	-42	-7	52
R Primary Somatosensorial Cortex, (BA 2)		11.13	51	-28	43		8.59	51	-25	43		12.56	51	-25	43
L Primary Somatosensorial Cortex, (BA 2)		8.46	-48	-34	46		9.70	-48	-34	46		7.22	-48	-34	46
R Primary Somatosensorial Cortex, (BA 3)		16.70	33	-34	67		13.91	39	-25	46		13.41	21	-37	58
L Primary Somatosensorial Cortex, (BA 3)		8.57	-24	-35	58		9.53	-24	-35	58		9.87	-24	-35	58
R Somatosensory Association Cortex, (BA 5)		15.29	27	-43	64		12.42	27	-46	64		11.88	27	-46	64
L Somatosensory Association Cortex, (BA 5)		13.83	-24	-43	67		11.18	-30	-43	64		8.38	-24	-43	67
R Parietal Lobe, Precuneus, (BA 7)		9.65	15	-46	52		9.55	12	-43	52		14.35	12	-43	52
L Parietal Lobe, Precuneus, (BA 7)		13.37	-12	-46	55		8.62	-12	-49	58		13.10	-12	-49	58
L Frontal Lobe, Paracentral Lobule, (BA 31)		9.51	3	-22	49		11.41	3	-22	49		7.49	3	-22	49
L Frontal Lobe, Paracentral Lobule, (BA 31)		14.42	-3	-22	49		10.36	-6	-22	46		7.75	-3	-22	49
R Parietal Lobe, Postcentral Gyrus, (BA40)		17.97	39	-37	61		11.96	36	-37	55		16.69	39	-31	43
L Parietal Lobe, Postcentral Gyrus, (BA40)		8.60	-39	-34	55		9.86	-39	-40	58		11.19	-39	-31	46
